# Aging retinal function is improved by near infrared light (670 nm) that is associated with corrected mitochondrial decline

**DOI:** 10.1016/j.neurobiolaging.2017.01.001

**Published:** 2017-04

**Authors:** Chrishne Sivapathasuntharam, Sobha Sivaprasad, Christopher Hogg, Glen Jeffery

**Affiliations:** a University College London Institute of Ophthalmology, London, UK; b Moorfields Eye Hospital, London, UK

**Keywords:** Retina, Aging, Mitochondria

## Abstract

Aging is associated with cellular decline and reduced function, partly mediated by mitochondrial compromise. However, aged mitochondrial function is corrected with near infrared light (670 nm) that improves their membrane potentials and adenosine triphosphate production and also reduces age-related inflammation. We ask if 670 nm light can also improve declining retinal function. Electroretinograms were measured in 2-, 7-, and 12-month old C57BL/6 mice. Significant age-related declines were measured in the photoreceptor generated a-wave and the postreceptoral b-wave. Seven- and 12-month-old mice were exposed to 670 nm for 15 minutes daily over 1 month. These showed significant improved retinal function in both waves of approximately 25% but did not reach levels found in 2-month-old animals. Our data suggest, 670 nm light can significantly improve aged retinal function, perhaps by providing additional adenosine triphosphate production for photoreceptor ion pumps or reduced aged inflammation. This may have implications for the treatment of retinal aging and age-related retinal disease, such as macular degeneration.

## 1 Introduction

Aging is associated with declining mitochondrial function, with reductions in mitochondrial membrane potentials and adenosine triphosphate production (ATP) (Gkotsi et al., 2014, Harman, 1972, Kokkinopoulos et al., 2013, Kujoth et al., 2005, Lane, 2005). The pace of aging is linked to metabolic rate, with high rates associated with faster aging (Speakman, 2005, Wang et al., 2010). The retina is a key example of this as photoreceptors have the greatest energy demand in the body (Linsenmeier and Padnick-Silver, 2000). Here, ATP declines significantly by 3–4 months in mice. At the same time, mitochondrial membrane potentials decline (Kokkinopoulos et al., 2013), chronic inflammation becomes established (Catchpole et al., 2013, Hoh Kam et al., 2013, Xu et al., 2009), and retinal function declines (Kolesnikov et al., 2010, Li et al., 2001). These events herald a 30% photoreceptor loss in both mouse and man (Cunea and Jeffery, 2007, Cunea et al., 2014, Curcio, 2001).

Some of these features can be corrected. Specific long wavelengths of light are absorbed by cytochrome c oxidase in mitochondria (Fitzgerald et al., 2013), and this is associated with improved respiration, increased membrane potentials and improved ATP production (Gkotsi et al., 2014, Kokkinopoulos et al., 2013), and reductions in key markers of age-related retinal inflammation (Begum et al., 2013). In Drosophila, long wavelength light also increases mean lifespan and mobility (Begum et al., 2015), and in bumble bees, it has similar impact but also improves the electroretinogram in normal animals and those in which mitochondrial function has been undermined by insectiside exposure (Powner et al., 2016). Here, we ask if 670 nm light exposure that is associated with corrected mitochondrial function and reduced inflammation translates to improved retinal function in aged mice.

## 2 Materials and methods

### 2.1 Mice

Thirty-five female C57BL/6 mice were used at 3 different ages (n = 4 at 2 months, n = 18 at 7 months, and n = 13 at 12 months). Mice at 7 and 12 months were divided into treated (7 months n = 8, 12 months n = 7) and untreated (7 months n = 10, 12 months n = 6). All animals were maintained under identical standard laboratory conditions. Experimental mice were exposed for 15 min/d to 670 nm light (40 mW/cm^2^, 36 J in total) via LEDs (CH Electronics, UK) at approximately 10 AM. Light exposures and light environment were similar to that in Begum et al. (2013) with background room lighting being approximately 2.256 × 10^−2^ W/m^2^.

### 2.2 Electrophysiology

Dark-adapted mice underwent full field scotopic (intensity sequence) and photopic (single flash, intensity sequence) ERG recordings (Diagnosys LLC, Cambridge, UK) after being anesthetized intraperitoneally with 6% ketamine (National Veterinary Services Ltd, UK), 10% Dormitor (National Veterinary Services Ltd, UK), and 84% sterile water at 5 μL/g. Pupils were dilated (1%Tropicamide, Bausch and Lomb, France), and the cornea lubricated with Viscotears (Novartis, Switzerland). Ground and reference electrodes were subdermal. ERGs were undertaken at increasing stimulus strengths using a 6500K white light at 2.5 × 10^−5^, 1.25 × 10^−4^, 1.14 × 10^−3^, 0.03, 0.32, 3.11, and 31.90 sct cd s m^−2^. After the scotopic series, mice were adapted to a 20 cd m^−2^, rod saturating background for 15 minutes. Photopic responses to single white light flash stimuli of 0.3, 2.8, 28.1, and 84.2 cd s m^−2^ were recorded with a background light of 20 cd m^−2^.

### 2.3 Immunohistochemistry

Mice were killed by cervical dislocation and eyes placed in 4% paraformaldehyde in phosphate buffered saline (PBS), pH 7.4 for 1 hour, cryopreserved in 30% sucrose and embedded in optimum mounting medium (Agar Scientific Ltd) and cryosectioned at 10 μm. Sections were incubated for 1 hour in 5% normal donkey serum (NDS) in 0.3% Triton X-100 in PBS, then incubated overnight with COX III (goat polyclonal 1:250) diluted in 1% NDS in 0.3% Triton X-100 in PBS. Negative controls had the primary antibody omitted. Sections were incubated for 1 hour in donkey anti-goat secondary antibody conjugated with Alexa Fluor 568 (1:2000, Invitrogen) diluted in 2% NDS in 0.3% Triton X-100 in PBS. The slides were mounted with Vectashield (VECTOR laboratories). Photographs of sections were taken at ×400 JPEG format and analyzed using Adobe Photoshop CS4 in the same manner as Begum et al. (2013).

Statistical analysis was undertaken with a 2-way analysis of variance for electrophysiological data between groups over progressive intensities and between groups at specific intensities (1-way analysis of variance). Statistics for the immunohistochemistry were undertaken with a 1-tailed Mann-Whitney *U* test.

## 3 Results

**Fig. 1:**
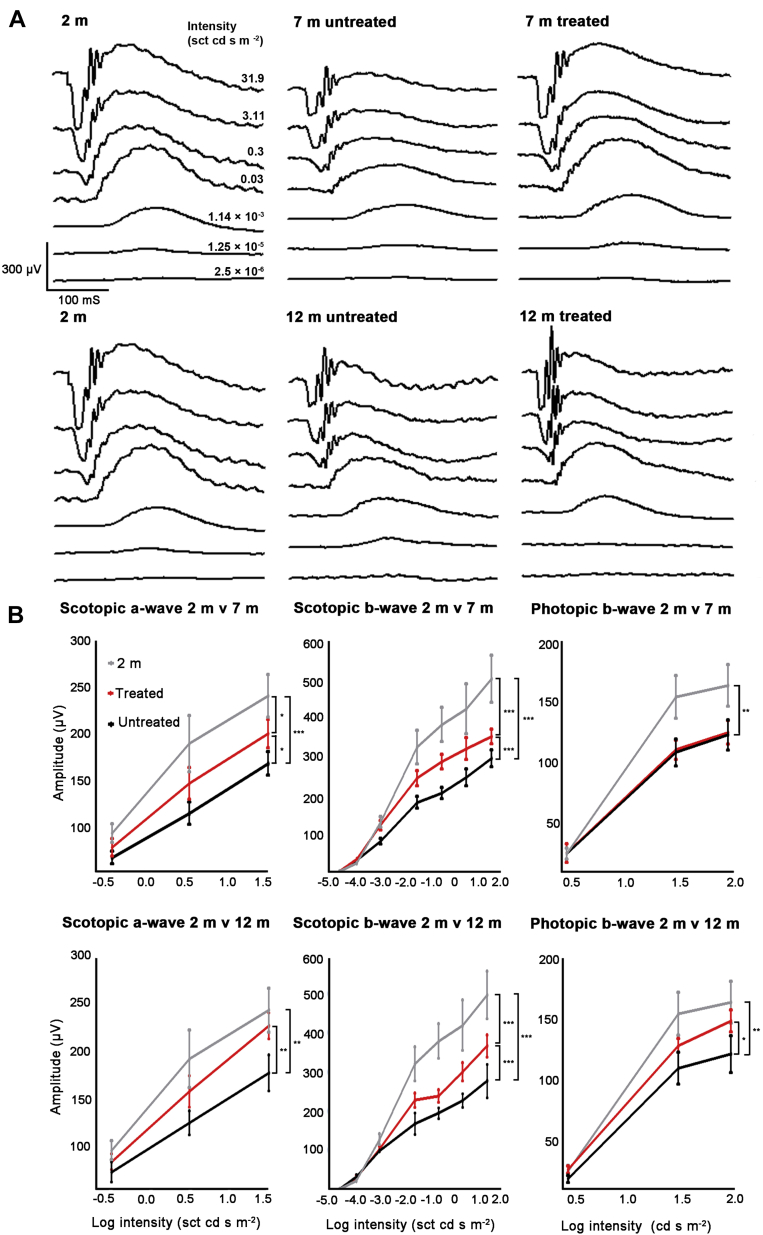
Examples of ERG recordings from 2-, 7-, and 12-month-old mice and the impact of treatment with 670 nm light. (A) Panel shows the waveforms of the ERGs. The waves from 2-month-old mice are shown twice, once in the first row with 7-month animals and again in the second row with 12-month animals for direct comparison. Reductions with age are apparent in untreated aged mice. Improvements on this are apparent in both treated groups in the right hand column. (B) Panel shows the analysis of waveforms with statistical comparisons of aged decline and improvements following 670 nm light treatment. Significant improvements in signal amplitude were found following treatment in all groups except for the photopic response at 7 months. Abbreviations and symbols: M, month. ^∗^*p* < 0.05, ^∗∗^*p* < 0.01, and ^∗∗∗^*p* < 0.001. Error bars are standard error of the mean. Analysis at progressive light intensities between the 3 groups (1 way ANOVA) revealed for scotopic a-wave at 2 m versus 7 m, NS for the first 2 intensities and *p* < 0.05 for the final. Scotopic b-wave 2 m versus 7 m NS for the first 3 intensities and *p* < 0.05 for the subsequent 4. Photopic b-wave 2 m versus 7 m each NS. Scotopic a-wave 2 m versus 12 m, first 2 intensities NS, final intensity *p* < 0.05. Scotopic b-wave 2 m versus 12 m, first 3 intensities NS, subsequent intensities all *p* < 0.05. Photopic b-wave 2 m versus 12 m, first 2 intensities *p* < 0.05, final intensity NS.

There are reductions in the a- and b-waves of the ERG and delays in timing with age (Birch and Anderson, 1992, Kolesnikov et al., 2010, Neveu et al., 2011). Fig. 1A shows example ERG traces from each of the groups of mice at increasing light intensities. Data from 2-month animals are represented twice alongside both 7- and 12-month animals for direct comparison. These show age-related reductions in amplitude and improvements with 670 nm treatment.

Fig. 1B shows that significant age-related differences are present in scotopic responses in 7- and 12-month old mice compared with 2-month old mice, with reductions in the magnitude of both negative a-wave (7 months *p* < 0.001, 12 months *p* < 0.01) and subsequent positive b-wave (7 months *p* < 0.001, 12 months *p* < 0.001). At higher intensities, reductions in the a-wave were approximately 20% in the 7-month animals and approximately 30% in the 12-month animals. b-wave reductions were approximately 40% in both groups. The photopic b-waves were reduced by approximately 25%. These differences were statistically significant (7 and 12 months both *p* < 0.001). Age-related timing differences were not significant.

Fig. 1A and B also show differences between treated and untreated mice. Scotopic responses in treated mice were generally increased by approximately 20% in the a-wave in both 7- and 12-month mice, which was significant (7 months *p* < 0.05; 12 months *p* < 0.01). However, these did not reach the amplitudes found at 2 months. There was a 15% difference between 2 months and both aged groups, which was significant at 7 months (*p* < 0.05) but not at 12 months, implying a greater improvement in 12-month treated mice. Statistical differences between the groups for the ERGs at progressive stimulus light intensity are given in the figure legend.

Significant improvements were also found in the b-wave of approximately 30% in the 7-month animals and 20% in the 12-month mice (both significant: 7 months and 12 months *p* < 0.001). But in both cases, improvements remained significantly different from 2 months responses by approximately 30% at higher intensities (7 months and 12 months *p* < 0.001). There were no timing differences. A significant improvement in the photopic b-wave was only found in 12-month mice of 20% (*p* < 0.05). No significant differences were found between these treated mice and 2-month-old animals, similar to the scotopic a-wave in treated 12 months mice. Again there were no timing differences.

**Fig. 2:**
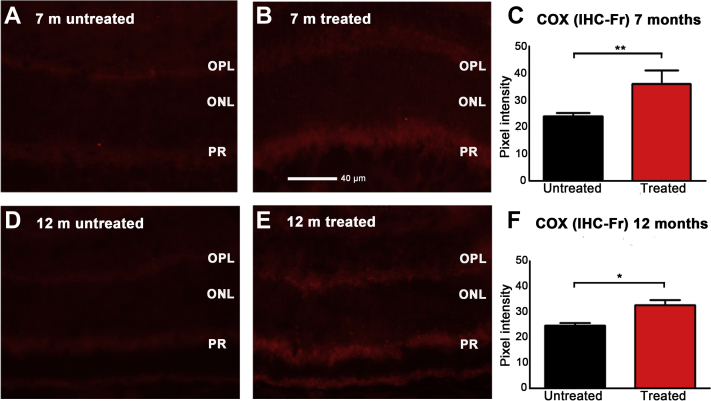
COX immunohistochemistry in experimental and untreated mice at 7 m (A and B). Higher levels were found in experimental animals compared to controls (C). Similar data at 12 m (D and E), which were again significantly different with greater levels in 670 nm exposed mice (F). ^∗^*p* < 0.05 and ^∗∗^*p* < 0.01. Abbreviations: IHC-Fr, immunohistochemistry frozen section; ONL, outer nuclear layer; OPL, outer plexiform layer; PR, photoreceptor inner segments.

Retinae stained for COX (Fig. 2) showed that in each of the aged groups, COX levels were significantly greater in mice exposed to 670 nm than in their age-matched unexposed controls at both 7 months (*p* < 0.01) and 12 months (*p* < 0.05) groups. This confirmed an association between improved mitochondrial and retinal function.

## 4 Discussion

Our results show that brief, daily 670 nm exposure over a month significantly improves both the photoreceptor-generated a-wave and the postreceptoral b-wave ERG, but that they do not completely mitigate the impact of aging. This mirrors findings where 670 nm light has been used to protect against light induced photoreceptor degeneration, significant protection was afforded, but this did not provide complete protection (Albarracin et al., 2011). However, ERGs are relatively crude, and are thought to be about 2 log units less sensitive than psychophysical responses (Ruseckaite et al., 2011). Hence, the magnitude of 670 nm on the aging retina may be greater than that revealed here. We did find significant improvements in photopic responses at 12 months but not at 7 months. Hence, our data are consistent with a notion that 670 nm has a greater effect when animals are older.

The reason for improvements in amplitude are unclear but may relate to increased ATP availability to Na^+^/K^+^ ATP pumps, as these pumps decline with age (de Lores Arnaiz and Ordieres, 2014). Alternatively, a general improvement in photoreceptor physiology may be due to reduced inflammation (Begum et al., 2013).

Although near infrared light is of therapeutic value in induced pathology (Fitzgerald et al., 2013), it has not been extensively used in aging. However, we know that it reduces age-related inflammation and retinal stress (Begum et al., 2013, Begum et al., 2015, Calaza et al., 2015, Kokkinopoulos et al., 2013). The mechanism of action may relate to its absorption by cytochrome c oxidase in the electron transport chain. Subsequent changes in the redox state of this may increase ATP, which declines significantly by 4 months of age (Calaza et al., 2015, Gkotsi et al., 2014). However, there may not be a single mechanism behind improvements when exposure is over a long period (Karu, 1999).

The cellular environment is different when tissue has suffered from induced pathology, where 670 nm has been used extensively (Fitzgerald et al., 2013) compared with that in aging. Aging is a chronic condition where mitochondria decline gradually. However, in both situations, the light appears to offer significant benefit without adverse effects. As such it may provide significant value in problems of general aging. Furthermore, as declining mitochondrial function is implicated in age-related macular degeneration (Terluk et al., 2015), use of 670 nm light, particularly in early stages of the disease, could provide a therapeutic route to reducing its impact in a situation where little or no alternative exist.

## Disclosure statement

The authors have no conflict of interest. Animals were used with University College London ethical committee approval under a Home Office animal project license. All animal procedures conformed to the United Kingdom Animals Scientific Procedures Act 1986.
